# Supplementary material for: Novel Driver Strength Index highlights important cancer genes in TCGA PanCanAtlas patients
Source: PeerJ. 2022 Aug 11;10:e13860. doi: 10.7717/peerj.13860 (PMC9375969; doi:10.7717/peerj.13860)
Supplement: Supplemental Information 2 [file peerj-10-13860-s002.zip › COHORTS/genes plots/age/2021_8_16_14_9_distribution_cohorts_males_70-74.pdf]

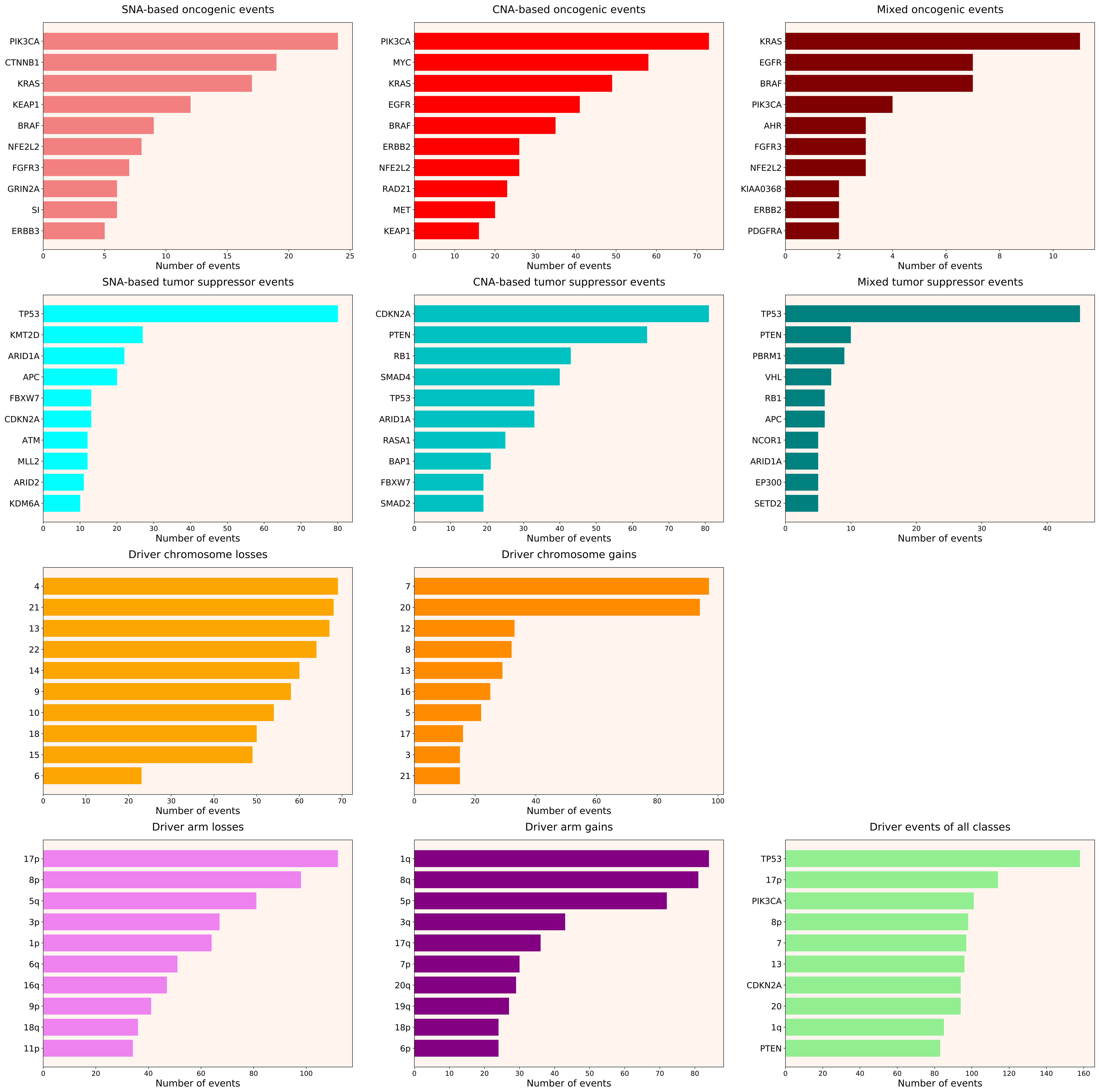

SNA-based oncogenic events
PIK3CA
CTNNB1
KRAS
KEAP1
BRAF
NFE2L2
FGFR3
GRIN2A
SI
ERBB3
0 5 10 15 20 25
Number of events
CNA-based oncogenic events
PIK3CA
MYC
KRAS
EGFR
BRAF
ERBB2
NFE2L2
RAD21
MET
KEAP1
0 10 20 30 40 50 60 70
Number of events
Mixed oncogenic events
KRAS
EGFR
BRAF
PIK3CA
AHR
FGFR3
NFE2L2
KIAA0368
ERBB2
PDGFRA
0 2 4 6 8 10
Number of events
SNA-based tumor suppressor events
TP53
KMT2D
ARID1A
APC
FBXW7
CDKN2A
ATM
MLL2
ARID2
KDM6A
0 10 20 30 40 50 60 70 80
Number of events
CNA-based tumor suppressor events
CDKN2A
PTEN
RB1
SMAD4
TP53
ARID1A
RASA1
BAP1
FBXW7
SMAD2
0 10 20 30 40 50 60 70 80
Number of events
Mixed tumor suppressor events
TP53
PTEN
PBRM1
VHL
RB1
APC
NCOR1
ARID1A
EP300
SETD2
0 10 20 30 40
Number of events
Driver chromosome losses
4
21
13
22
14
9
10
18
15
6
0 10 20 30 40 50 60 70
Number of events
Driver chromosome gains
7
20
12
8
13
16
5
17
3
21
0 20 40 60 80 100
Number of events
Driver arm losses
17p
8p
5q
3p
1p
6q
16q
9p
18q
11p
0 20 40 60 80 100
Number of events
Driver arm gains
1q
8q
5p
3q
17q
7p
20q
19q
18p
6p
0 10 20 30 40 50 60 70 80
Number of events
Driver events of all classes
TP53
17p
PIK3CA
8p
7
13
CDKN2A
20
1q
PTEN
0 20 40 60 80 100 120 140 160
Number of events
